# Accelerating the convergence of Newton's method for the Yang-Baxter like matrix equation

**DOI:** 10.1016/j.heliyon.2025.e42425

**Published:** 2025-02-03

**Authors:** Chacha Stephen Chacha

**Affiliations:** Department of Mathematics, Physics and Informatics, Mkwawa University College of Education, P.O. Box 2513, Iringa, Tanzania

**Keywords:** Matrix equation, Normwise, mixed, and componentwise condition numbers, Exact line search, Successive over-relaxation

## Abstract

This article explores the application of exact line search and successive over-relaxation techniques to enhance the convergence of Newton method in solving the Yang-Baxter matrix equation for nontrivial numerical solutions. Moreover, the normwise, mixed, and componentwise condition numbers are derived to assess the sensitivity of solutions. Numerical experiments demonstrate that the exact line search method significantly improves convergence speed, particularly for larger matrices, by reducing both the number of iterations and residuals more effectively than the successive over-relaxation technique. Furthermore, the mixed and componentwise condition numbers consistently yield values close to one, indicating that the Yang-Baxter equation is well-conditioned. In contrast, the relatively high normwise condition numbers suggest an increased sensitivity to perturbations.

## 1 Introduction

Nonlinear matrix equations appear in numerous applications. A significant category of these equations, found in control theory, are Riccati equations. The study of Riccati equations and the development of numerical methods for their solution are well-established [1], [2]. In this article, we consider the Yang-Baxter-like matrix equation (YBME), which is stated as

(1) F(X)=AXA−XAX.

The YBME draws its inspiration from the Yang-Baxter equation (YBE) in statistical mechanics, originally developed by Yang and Baxter [3], [4]. This equation plays a pivotal role in understanding integrable systems, quantum groups, and low-dimensional topology [5]. The YBE has applications across various scientific and engineering disciplines [26]. By exploring its matrix counterpart, we seek to apply the profound insights from these fields to the study of matrix equations. The YBME has recently garnered significant attention in the fields of matrix theory, numerical linear algebra, and functional analysis [7], [20], [21], [22], [23]. Given its theoretical roots in integrable systems, solutions to this equation might reveal underlying symmetries and invariants that are otherwise not apparent, leading to more efficient computational approaches and a deeper understanding of matrix dynamics [24], [25].

Previous research has investigated different non-trivial solutions of the YBME. Adam explored commuting and non-commuting solutions, deriving conditions for the latter under a specific full-rank condition [8]. Chen investigated explicit solutions for diagonalizable matrices, while Kumar explored explicit solutions for YBM, offering insights into numerical computation [9], [10]. Studies have also addressed specific cases, such as rank-one matrices [7] and permutation matrices [11]. Additionally, there has been comprehensive exploration of rank formulas for the YBME, as illustrated by the in-depth investigation presented in [12]. Iterative methods are a key approach in solving the Yang-Baxter matrix equation, which is a complex system of non-linear matrix equations. While much of the focus in existing literature has been on understanding the structure of these matrix equations, the survey of iterative methods specifically for solving the Yang-Baxter equation remains insufficient. Therefore, an extensive review of these methods, particularly in relation to nonlinear systems, is necessary to advance this area of research. Notable works in this domain include the HSS-like method for complex nonlinear Yang-Baxter matrix equations, which provides an efficient solution approach [42] and iterative schemes for generalized Sylvester matrix equations that are also applicable to Yang-Baxter systems [43]. Additionally, approximating optimal parameters for generalized preconditioned Hermitian and skew-Hermitian splitting methods [44] and generalized product-type methods based on bi-conjugate gradient methods [45] have shown promise in addressing such nonlinear systems.

Recently, [6] presented an efficient numerical method for finding a nontrivial symmetric solvent of the YBME. Additionally, [32] provided a unique zeroing neural network model and an efficient matrix numerical method for deriving numerical commuting solvents of the time-invariant YBME. The suggested approach is shown to be stable and has a second-order convergence speed.

In this paper, we build upon previous studies by introducing exact line searches and successive over-relaxation techniques to accelerate the Newton method for finding the nontrivial iterative solvent of the YBME for real matrix coefficient. When the coefficient matrix contains complex entries, the methods proposed in [46], [47] provide effective solutions. We also investigate the numerical stability and reliability of the YBME solution through a detailed analysis of normwise, mixed, and componentwise condition numbers, providing valuable insights into the conditioning of the problem. Condition numbers play a crucial role in numerical analysis by measuring the sensitivity of mathematical problems to input perturbations. Their significance extends to numerous applications, including control theory and data science, where solution stability is paramount. The foundational framework for condition numbers was established by Rice [35], who introduced their theoretical basis. This work was later expanded by Gohberg and Koltracht [36], who explored mixed, componentwise, and structured condition numbers for matrix problems. Subsequent contributions further enriched the field. Diao, Xiang, and Wei [34] introduced statistical condition estimation for Sylvester equations, while Samar, Li, and Wei [33] examined condition numbers for the K-weighted pseudoinverse. Wang, Yang, and Li [37] addressed nonlinear matrix equations, and Samar, Zhu, and Xu [38] developed conditioning theory for ML-weighted pseudoinverses. Moreover, Li, Wang, and Zheng [39] investigated periodic Sylvester equations, and Samar and Lin [40] focused on the condition numbers of Tikhonov regularization for total least squares. These studies collectively highlight the critical role of condition numbers in ensuring numerical stability and reliability. While normwise condition numbers provide a broad measure of sensitivity, their effectiveness can be limited in ill-conditioned problems or when stability depends significantly on the choice of norm [17]. To address such limitations, componentwise analyses such as mixed and componentwise condition numbers provide more nuanced stability assessments [13], [15]. By capturing detailed problem-specific sensitivities, these approaches improve our understanding of the underlying numerical behavior.

The rest of this article is organized as follows: In Section 2, we provide useful definitions, notations, and lemmas that will be used in subsequent sections. Section 3 presents the derivation of Newton method, along with exact line search and successive overrelaxation techniques for solving equation (1). In Section 4, we derive explicit expressions for normwise, mixed, and componentwise condition numbers. Section 5 presents three numerical examples to illustrate the proposed methods. Finally, a brief conclusion is given in Section 6.

## 2 Preliminaries

We present essential notations and definitions crucial for our proofs. The symbol $A^{T}$ represents the transpose of matrix A. The Frobenius norm of a matrix A, denoted by ${\left\|A\right\|}_{\text{F}}$, is defined as the square root of the sum of the squares of all its entries, i.e.,

$${\left\|A\right\|}_{\text{F}}={\left(\sum_{i=1}^{m}\sum_{j=1}^{n}a_{ij}^{2}\right)}^{1/2},$$

where $a_{ij}$ are the elements of A. For $A_{1}=(a_{ij})\in C^{n_{A}\times m_{A}}$ and $B_{1}\in C^{n_{B}\times m_{B}}$, the tensor product is defined as

$$A_{1}⊗B_{1}=\left(\begin{array}{cccc} a_{11}B_{1} & a_{12}B_{1} & \cdots & a_{1m_{A}}B_{1}\\ a_{21}B_{1} & a_{22}B_{1} & \cdots & a_{2m_{A}}B_{1}\\ \vdots & \vdots & \ddots & \vdots \\ a_{n_{A}1}B_{1} & a_{n_{A}2}B_{1} & \cdots & a_{n_{A}m_{A}}B_{1} \end{array}\right)\in C^{\left(n_{A}n_{B})\times (m_{A}m_{B}\right)}.$$

The maximum norm of a matrix A, denoted by ${\left\|A\right\|}_{\max}$, is defined as the largest absolute value among its entries. Mathematically, it is given by:

$${\left\|A\right\|}_{\max}=\max_{i,j}|a_{ij}|,$$

where $a_{ij}$ represents the (i,j)-th element of the matrix A. The infinity norm of a matrix A, denoted by ${\left\|A\right\|}_{\infty }$, is defined as the maximum absolute row sum of the matrix. It is expressed as:

$${\left\|A\right\|}_{\infty }=\max_{1\leq i\leq m}\sum_{j=1}^{n}|a_{ij}|,$$

where *m* and *n* are the dimensions of A, and $\left|a_{ij}\right|$ is the absolute value of the (i,j)-th entry. The notation |i,j〉 represents a quantum state in the tensor product Hilbert space $H_{1}⊗H_{2}$, where *i* and *j* are indices labeling the basis states of the first and second subsystems, respectively. In the context of the spin-1 Heisenberg chain, *i* corresponds to the spin quantum number $m_{S}^{\left(1\right)}$ of the first particle, and *j* corresponds to $m_{S}^{\left(2\right)}$ of the second particle. Each index i,j∈{1,2,3} maps to the eigenvalues $m_{S}\in \{-1,0,1\}$.

Definition 1 Consider a matrix $A\in R^{m\times n}$ with columns $a_{i}\in R^{m}$ for i=1,2,…,n. The vector vec[A] is expressed as an *mn*-dimensional vector formed by stacking the columns of *A*, given by

$$\text{vec}[A]=\left[\begin{array}{c} a_{1}\\ a_{2}\\ \vdots \\ a_{n} \end{array}\right]\in R^{mn}.$$

For matrices *A*, *B*, and *X*, one can observe that:

$$\text{vec}[AXB]=(B^{T}⊗A)\text{vec}[X].$$

Definition 2 The Arithmetic Mean-Geometric Mean (AM-GM) inequality states that for any non-negative real numbers *a* and *b*, the following inequality holds:

$$\frac{a+b}{2}\geq \sqrt{ab},$$

with equality if and only if a=b. This inequality asserts that the arithmetic mean is always greater than or equal to the geometric mean.

Definition 3 [41] The Fréchet derivative of a function $f:R^{m\times n}\to R^{p\times q}$ at a point $X\in R^{m\times n}$, denoted as Df(X), is defined by the limit:

$$Df(X)[H]=\lim_{\epsilon \to 0}\frac{f(X+\epsilon H)-f(X)}{\epsilon },$$

where $H\in R^{m\times n}$ is a perturbation matrix.

Definition 4 [41] Let $f:R^{m\times m}\to R^{m\times m}$ be a matrix function. The Fréchet derivative of the matrix function *f* at *X* in the direction *H* is denoted by the unique linear operator $L_{f}$, which maps *H* to $L_{f}(X,H)$, satisfying the relation

$$f(X+H)-f(X)=L_{f}(X,H)+O({\left\|H\right\|}^{2}),\;\;\forall \;\;X,\;H\in R^{m\times m}.$$

To introduce and componentwise and mixed condition numbers, we define a distance function for vectors $f,g\in R^{n}$. Let $f/g=[h_{1},h_{2},\cdots ,h_{n}]$ with components defined as

$$h_{i}=\left\{ \begin{array}{cc} \frac{f_{i}}{g_{i}}, & \text{if }g_{i}\neq 0,\\ 0, & \text{if }f_{i}=g_{i}=0,\\ \infty , & \text{otherwise}. \end{array}\right.$$

We proceed to establish that $d(f,g)={\left\|(f-g)/g\right\|}_{\infty }=\max_{\left(k=1,\;2,\;3,\;\cdots ,\;n\right)}\left\{\left|\frac{\left|f_{k}-g_{k}\right|}{\left|g_{k}\right|}\right|\right\}$, where d(f,g)<∞ allows extension of *d* to matrices, specifically for matrices F,G, as d(F,G)=d(vec(F),vec(G)). For a positive *ζ*, we denote $B_{\zeta }^{0}=\{x:d(a,x)\leq \zeta \}$.

Definition 5 [33], [34] Let $F:R^{p}\to R^{q}$ be a continuous mapping defined on an open set $\text{Dom}(F)\subset R^{p}$ and a∈Dom(F) satisfy a≠0 and F(a)≠0.

*(i)* The *normwise condition number* of F at *a* is defined by
  $$\kappa (F,a)=\lim_{\varepsilon \to 0}\underset{\begin{array}{c} x\in B(a,\varepsilon )\\ x\neq a \end{array}}{\sup}\left(\frac{{\left\|F(x)-F(a)\right\|}_{2}}{{\left\|F(a)\right\|}_{2}}/\frac{{\left\|x-a\right\|}_{2}}{{\left\|a\right\|}_{2}}\right).$$
*(ii)* The *mixed condition number* of F at *a* is defined by
  $$m(F,a)=\lim_{\varepsilon \to 0}\underset{\begin{array}{c} x\in B^{0}(a,\varepsilon )\\ x\neq a \end{array}}{\sup}\frac{{\left\|F(x)-F(a)\right\|}_{\infty }}{{\left\|F(a)\right\|}_{\infty }}\cdot \frac{1}{d(x,a)}.$$
*(iii)* The *componentwise condition number* of F at *a* is defined by
  $$c(F,a)=\lim_{\varepsilon \to 0}\underset{\begin{array}{c} x\in B^{0}(a,\varepsilon )\\ x\neq a \end{array}}{\sup}\frac{\left(F(x),F(a)\right)}{d(x,a)}.$$

Lemma 1 leverages the Fréchet derivative to provide explicit expressions for the normwise, mixed, and componentwise condition numbers.

Lemma 1 **[33]**

*Under the same assumptions as in*Definition 5*, if*F*is Fréchet differentiable at a, then the condition numbers are given by*

$$\kappa (F,a)=\frac{{\left\|\text{d}F(a)\right\|}_{2}{\left\|a\right\|}_{2}}{{\left\|F(a)\right\|}_{2}},\;m(F,a)=\frac{{\left\||\text{d}F(a)||a|\right\|}_{\infty }}{{\left\|F(a)\right\|}_{\infty }},\;c(F,a)={\left\|\frac{\left|\text{d}F(a)||a\right|}{\left|F(a)\right|}\right\|}_{\infty },$$

*where*dF(a)*denotes the Fréchet derivative of*F*at a.*

## 3 Newton's method for obtaining the solution of Eq. (1)

In this section, we derive Newton's method for solving the equation (1). Newton's method is a classical technique for finding roots of real-valued functions, and we extend this approach to matrix equations. To develop the iterative procedure at the core of Newton's method, we analyze the behavior of the function F at X+H, where *H* represents a perturbation or correction term applied to the current approximation *X*. By employing the Fréchet derivative of F with respect to *X*, we obtain the necessary iterative formula. Additionally, we introduce enhancements such as exact line searches and successive relaxation techniques to improve the convergence speed and stability of the method. These refinements are particularly valuable when the initial guess is far from the true solution, as they accelerate convergence and improve the algorithm's robustness.

From Eq. (1), we have

(2) $$F(X+H)=A(X+H)A-(X+H)A(X+H)=F(X)+F_{X}^{\prime }(H)-HAH=F(X)+F_{X}^{\prime }(H)+O({\left\|H\right\|}^{2}).$$

Considering the Fréchet derivative as a linear operator, $F_{X}^{\prime }(H):C^{n\times n}\to C^{n\times n}$ is defined by:

(3) $$F_{X}^{\prime }(H)=AHA-XAH-HAX.$$

Employing the vec operator to (3), we get:

(4) $$\mathrm{vec}(F_{X}^{\prime }(H))=D_{X}\mathrm{vec}(H),$$

where $D_{X}=A^{T}⊗A-I⊗\left(XA\right)-{\left(XA\right)}^{T}⊗I$ is the tensor Fréchet derivative of F(X). If $A^{T}⊗A-I⊗\left(XA\right)-{\left(XA\right)}^{T}⊗I$ is non-singular, then a Newton step is computed in the iteration:

(5) $$F_{X}^{\prime }(H)=-F(X)$$

and the solvent of (1) can be obtained by iteration (6):

(6) $$X(i+1)=X(i)-{\left[F_{X(i)}^{\prime }\right]}^{-1}F(X(i))\;\forall i=0,\;1,\;2,\cdots .$$

This iterative process aims to find the matrix *X* that satisfies F(X)=0. The analysis above lead to Method 1.

Method 1: Pure Newton method with no exact line search

1. Start with a matrix $A\in R^{n\times n}$ and initial starting point $X(0)\in R^{n\times n}$.
2. Determine H(k) by solving generalized Lyapunov equation (5).
3. Update the iteration using X(k+1)=X(k)+H(k) for k=0,1,2,⋯.
4. Verify if the norm ${\left\|X(k+1)-X(k)\right\|}_{\text{F}}\leq \epsilon$. Proceed to step 2 if this condition is not met, and stop otherwise.
5. Print the solution *X*, the computation time in seconds, and the residual ${\left\|F(X(k))\right\|}_{\text{F}}$.

Incorporating line searches: For solving non-constrained optimization problems, quasi-Newton methods typically employ the Newton direction as the search direction. The objective function is then minimized along this direction, either precisely or approximately, to define the next iteration [28]; this minimization is known as a line search. Benner and Byers [18] (see also [19]) analyze how Newton's method for solving quadratic equations incorporates exact line searches. Also, [14] employed exact line search technique to solve the nonlinear matrix equation. We employ exact line searches technique with iteration (6) for the YBME. Line searches are motivated by the idea that the Newton step *H* may not always be optimal and that the linear model of F(X), which underpins Newton's method, is not always accurate. The purpose of line searches is to improve global convergence, ensuring convergence from arbitrary initial positions. To exemplify the concept, consult [19] for an example. Line searches are a well-established strategy in optimization [27]. The idea is to replace step 3 in Method 1 with the update $X_{j+1}=X_{j}+\lambda_{j}H_{j}$, where $\lambda_{j}$ is the step length. This step length is chosen to minimize ${\left\|F(X+\lambda_{j}H_{j})\right\|}_{\text{F}}^{2}$.

From (1), we obtain

(7) $$F(X+\lambda_{j}H_{j})=(X+\lambda_{j}H_{j})A(X+\lambda_{j}H_{j})-A(X+\lambda_{j}H_{j})A.$$

Expanding equation (7) and simplifying using definitions in equations (3) and (5), we get

(8) $$F(X+\lambda_{j}H_{j})=(X+\lambda_{j}H_{j})A(X+\lambda_{j}H_{j})-A(X+\lambda_{j}H_{j})A.=XAX+XA\lambda_{j}H_{j}+\lambda_{j}H_{j}AX+\lambda_{j}^{2}H_{j}AH_{j}-AXA-A\lambda_{j}H_{j}A=XAX-AXA+\lambda_{j}(XAH_{j}+H_{j}AX-AH_{j}A)+\lambda_{j}^{2}H_{j}AH_{j}=F(X)+\lambda_{j}F_{X}^{\prime }(H)+\lambda_{j}^{2}H_{j}AH_{j}.=(1-\lambda_{j})F(X)+\lambda_{j}^{2}H_{j}AH_{j}.$$

Thus, computing $\lambda_{j}$ to reduce ${\left\|F(X_{j+1})\right\|}_{\text{F}}$ is comparable to reducing the polynomial

(9) $$f_{j}(\lambda )=\text{trace}\left({\left((1-\lambda )F(X)+\lambda^{2}HAH\right)}^{⁎}\left((1-\lambda )F(X)+\lambda^{2}HAH\right)\right).$$

Let C=(1−λ)F(X) and $B=\lambda^{2}HCH$. Then, we have:

(10) $${\left(C+B\right)}^{⁎}(C+B)=C^{⁎}C+C^{⁎}B+B^{⁎}C+B^{⁎}B.$$

Substituting *C* and *B* back into Eq. (10) yields:

(11) $${\left((1-\lambda )F(X)+\lambda^{2}HAH\right)}^{⁎}\left((1-\lambda )F(X)+\lambda^{2}HAH\right)={\left((1-\lambda )F(X)\right)}^{⁎}(1-\lambda )F(X)+{\left((1-\lambda )F(X)\right)}^{⁎}\lambda^{2}HAH\;\;+{\left(\lambda^{2}HAH\right)}^{⁎}(1-\lambda )F(X)+{\left(\lambda^{2}HAH\right)}^{⁎}\lambda^{2}HAH.$$

Now, calculating each term in Eq. (11), we get Eq. (12):

(12) $${\left((1-\lambda )F(X)\right)}^{⁎}(1-\lambda )F(X)={\left(1-\lambda \right)}^{2}F{\left(X\right)}^{⁎}F(X){\left((1-\lambda )F(X)\right)}^{⁎}\lambda^{2}HAH=\lambda^{2}(1-\lambda )F{\left(X\right)}^{⁎}HAH{\left(\lambda^{2}HAH\right)}^{⁎}(1-\lambda )F(X)=\lambda^{2}(1-\lambda ){\left(HAH\right)}^{⁎}F(X){\left(\lambda^{2}HAH\right)}^{⁎}\lambda^{2}HAH=\lambda^{4}{\left(HAH\right)}^{⁎}HAH.$$

Substituting all the terms back into Eq. (10) gives:

(13) $${\left((1-\lambda )F(X)+\lambda^{2}HAH\right)}^{⁎}\left((1-\lambda )F(X)+\lambda^{2}HAH\right)={\left(1-\lambda \right)}^{2}F{\left(X\right)}^{⁎}F(X)+\lambda^{2}(1-\lambda )F{\left(X\right)}^{⁎}HAH\;\;+\lambda^{2}(1-\lambda )H^{⁎}A^{⁎}H^{⁎}F(X)+\lambda^{4}{\left(HAH\right)}^{⁎}HAH.$$

Taking the trace of both sides of Eq. (13) gives:

(14) $$\text{trace}\left({\left((1-\lambda )F(X)+\lambda^{2}HAH\right)}^{⁎}\left((1-\lambda )F(X)+\lambda^{2}HAH\right)\right)=\text{trace}\left({\left(1-\lambda \right)}^{2}F{\left(X\right)}^{⁎}F(X)\right)+\text{trace}\left(\lambda^{2}(1-\lambda )F{\left(X\right)}^{⁎}HAH\right)\;+\text{trace}\left(\lambda^{2}(1-\lambda )H^{⁎}A^{⁎}H^{⁎}F(X)\right)+\text{trace}\left(\lambda^{4}{\left(HAH\right)}^{⁎}HAH\right).$$

In Eq. (14), using the property that the trace of a scalar multiple is the scalar multiple of the trace, we obtain Eq. (15):

(15) $$\text{trace}\left({\left((1-\lambda )F(X)+\lambda^{2}HAH\right)}^{⁎}\left((1-\lambda )F(X)+\lambda^{2}HAH\right)\right)={\left(1-\lambda \right)}^{2}\text{trace}\left(F{\left(X\right)}^{⁎}F(X)\right)+\lambda^{2}(1-\lambda )\text{trace}\left(F{\left(X\right)}^{⁎}HAH\right)+\lambda^{2}(1-\lambda )\text{trace}\left(H^{⁎}A^{⁎}H^{⁎}F(X)\right)+\lambda^{4}\text{trace}\left({\left(HAH\right)}^{⁎}HAH\right).$$

From Eq. (15) and using the property $\text{trace}(Q^{⁎}Q)={\left\|Q\right\|}_{\text{F}}^{2}$, we obtain:

(16) $$f_{j}(\lambda )={\left(1-\lambda \right)}^{2}{\left\|F(X)\right\|}_{\text{F}}^{2}+\lambda^{2}(1-\lambda )\text{trace}\left(F{\left(X\right)}^{⁎}HAH\right)+\lambda^{2}(1-\lambda )\text{trace}\left(H^{⁎}A^{⁎}H^{⁎}F(X)\right)+\lambda^{4}{\left\|HAH\right\|}_{\text{F}}^{2}.$$

By letting

$$a={\left\|F(X)\right\|}_{\text{F}}^{2},\;b={\left\|HAH\right\|}_{\text{F}}^{2},$$

and

(17) $$c=\text{trace}\left(F{\left(X\right)}^{⁎}HAH^{⁎}\right)+\text{trace}\left(H^{⁎}A^{⁎}H^{⁎}F(X)\right).$$

It follows from (16) that

(18) $$f_{j}(\lambda )={\left(1-\lambda \right)}^{2}a+\lambda^{2}(1-\lambda )c+\lambda^{4}b.$$

By applying the inequality

$$|\text{trace}(M^{⁎}N)|\leq {\left\|M\right\|}_{F}{\left\|N\right\|}_{F},$$

where *M* and *N* are matrices, and using the Cauchy-Schwarz inequality for the trace inner product, we can derive the following expression for (17):

$$|c|\leq {\left\|F(X)\right\|}_{F}{\left\|HAH^{⁎}\right\|}_{F}+{\left\|H^{⁎}A^{⁎}H^{⁎}\right\|}_{F}{\left\|F(X)\right\|}_{F}.$$

Expanding Eq. (18), we get:

(19) $$f_{j}(\lambda )=a-2a\lambda +(a+c)\lambda^{2}-c\lambda^{3}+b\lambda^{4}.$$

In Eq. (19), if b=0, Eq. (20).

(20) $$f_{j}(\lambda )={\left(1-\lambda \right)}^{2}a$$

achieves its minimum value globally at λ=1, corresponding to the classical Newton step. When a=0, then *X* represents the solution. Hence, we assume b>0 and a>0. We aim to identify the global minimum of *f*, which can have at most two minima, one of which is the global minimum.

To achieve the main results, we first derived the first derivative of the given function $f_{j}(\lambda )$ with respect to *λ*. We then evaluated the derivative at specific points, λ=0 and λ=2, to examine its sign. For λ=0, the derivative simplifies to

(21) $$f_{j}^{\prime }(0)=-2a,$$

which is negative since a>0. For λ=2, we obtained

$$f_{j}^{\prime }(2)=2a-8c+32b.$$

By applying the definitions of *a*, *b*, and *c*, along with the Cauchy-Schwarz and AM-GM inequalities, it follows that 2a−8c+32b>0. This inequality holds because the positive terms 2*a* and 32*b* dominate the potentially negative term −8c, given that $c\leq 2\sqrt{ab}$. Thus, $f_{j}^{\prime }(2)>0$. This confirms that $f_{j}^{\prime }(0)<0$ and $f_{j}^{\prime }(2)>0$, fulfilling the required conditions.

Given $f_{j}^{\prime }(0)<0$ and $f_{j}^{\prime }(2)>0$, the derivative $f^{\prime }$ has a real root in (0,2], which corresponds to either the inflection point or a minimum of *f*. It is clear that when λ=1 we have a pure Newton step, thus it is wise to concentrate on the interval [0,2]. However, when the point is far from a solution, there is no guarantee that *f* will have a minimum in this interval.

Consequently, we define *λ* as:

(22) $$f(\lambda )=\min_{x\in [0,2]}f(x).$$

We need to examine two possible cases:

1. The intended global minimum is located at the real root within the interval (0,2), since the derivative $f^{\prime }$ has a single real root and a pair of complex conjugate roots.

2. If $f^{\prime }$ has three real zeros, the minimum value of *f* can occur at only two of them. If the global minimum lies outside (0,2), it is necessary to check λ=2, as it may yield a value of *f* smaller than that at the real root of $f^{\prime }$ within (0,2).

Given the aforementioned conditions, determining *λ* in Eq. (22) is straightforward as it involves computing *f* values at these zeros and at the cubic roots of $f^{\prime }$. Exact line searches do not necessarily hinder the convergence of quadratic Newton's method, highlighting the importance of explicitly setting λ=1 as convergence is reached. As will be shown later, this is generally achievable under reasonable assumptions.

Suppose $X_{j}$ lies within the interval where quadratic convergence to *X* occurs. Let $X_{j+1}=X_{j}+H_{j}$ represent the pure Newton update, and ${\overset{ˆ}{X}}_{j+1}=X_{j}+\lambda H_{j}$ denote the update using exact line search. Let $\delta_{j}=X-X_{j}$, then we have:

$$O(\|\delta_{j+1}\|)=O({\left\|\delta_{j}\right\|}^{2}).$$

The way that *λ* is defined guarantees that, using Eq. (8).

(23) $$\|(1-\lambda_{j})F(X_{j})+\lambda_{j}^{2}H_{j}AH_{j}\|=\|(F(X_{j})+\lambda_{j}H_{j}\|\leq \|(F(X_{j})+H_{j}\|,=\|F(X_{j+1})\|=\|F(X-\delta_{j+1})\|,=O(\|\delta_{j+1}\|)+\|F(X)\|,=O({\left\|\delta_{j+1}\right\|}^{2}).$$

We then have $H_{j}=-\delta_{j+1}+\delta_{j}$, so $\left\|H_{j}\|=O(\|\delta_{j}\|\right)$ and by Eq. (2).

$$F(X_{j})=F(X-\delta_{j})=F(X)(\delta_{j})+O({\left\|\delta_{j}\right\|}^{2}).$$

Therefore, provided that the Fréchet derivative at *X* is invertible, Eq. (23) suggests that $\left|1-\lambda |=O(\|\delta_{j}\|\right)$. Thus,

$$X-{\overset{ˆ}{X}}_{j+1}=O({\left\|\delta_{j}\right\|}^{2})+(1-\lambda )H_{j}=X-X_{j+1}+X_{j+1}-{\overset{ˆ}{X}}_{j+1}=O({\left\|\delta_{j}\right\|}^{2}),$$

as required. Standard theory provides the global convergence qualities of Newton's method with accurate line searches. Newton's technique, where $g:R^{n^{2}}\mapsto R^{n^{2}}$, successfully solves the nonlinear system g(x)=0. Wang et al. [29] recommend using line searches on the function $F(x)=g{\left(x\right)}^{T}g(x)$. The global convergence findings in [31] and [30] hold true as long as the line search is subject to a set of limitations known as the Armijo-Goldstein requirements. These conditions can be expressed in our notation as

(24a) $$f(\lambda )\leq f(0)+\rho_{1}\lambda f^{\prime }(0),$$

(24b) $$f^{\prime }(\lambda )\geq \rho_{2}f^{\prime }(0).$$

Here, $\rho_{1}$ and $\rho_{2}$ are parameters such that $0<\rho_{1}<\rho_{2}<1$. The first condition guarantees that the reduction in *f* is at least as significant as predicted by a first-order model. The second condition ensures that the step size is not excessively small by requiring that the derivative at *λ* is at least a certain fraction of the derivative at zero. It is evident from Eq. (21) that Eq. (24a) is equivalent to

$$\|F(X+\lambda H)\|\leq (1-2\rho_{1}\lambda ){\left\|F(X)\right\|}_{\text{F}}^{2},$$

which necessitates adequate reduction in *f*. While exact line searches do not necessarily ensure that conditions (24a), (24b) are met, Eq. (24b) holds in the usual case when the optimal *λ* is a root of $f^{\prime }(\lambda )$, given that $f^{\prime }(0)<0$. The analysis above leads us to the Method 2.

Method 2: Pure Newton method with exact line search

1. Input a matrix $A\in R^{n\times n}$ and an initial guess $X(0)\in R^{n\times n}$.
2. Solve for H(k) in the Lyapunov Eq. (5).
3. Find a local optimizer λ(k)∈[0,2] in
  $$f(\lambda )={\left\|F(X(k)+\lambda H(k))\right\|}_{\text{F}}^{2}.$$
4. Update the iteration using X(k+1)=X(k)+λ(k)H(k) for all k=0,1,2,⋯.
5. Check if ${\left\|X(k+1)-X(k)\right\|}_{\text{F}}\leq \epsilon$. If true, stop; otherwise, go back to step 2.
6. Display the solution *X*, time in seconds and the residual ${\left\|F(X(k))\right\|}_{\text{F}}$.

To compute the optimal value of *λ* that minimizes a given objective function, we employ a numerical optimization approach using the fminsearch algorithm. The objective function is defined as

$$f(\lambda )={\left\|F(\lambda )\right\|}_{\text{F}}^{2},$$

where F(λ)=(X+λH)A(X+λH)−A(X+λH)A. The goal is to find *λ* such that f(λ) is minimized, which involves solving a nonlinear, scalar optimization problem. The optimization is performed using the fminsearch function, which utilizes the Nelder-Mead simplex algorithm. This is a derivative-free method that iteratively adjusts the value of *λ* to converge to a local minimum of f(λ). The process starts with an initial guess, here chosen as λ=1, and iteratively updates *λ* by evaluating the function at various points to identify a minimum. This approach is particularly advantageous for problems where the objective function is non-smooth or its derivatives are difficult to compute. While the method is computationally efficient for small-scale problems and ensures local convergence, it is limited to finding local minima and may require multiple initial guesses or supplementary techniques to explore the global solution space if the function is nonconvex. This numerical strategy provides a practical and flexible framework for solving scalar optimization problems in the absence of closed-form solutions or gradient information.

To incorporate the successive overrelaxation (SOR) into Method 1, we need to adjust the iterative update step to include an overrelaxation parameter, which helps accelerate the convergence of iterative process. The parameter ω∈(0,2)∖{1} is the *overrelaxation parameter* introduced in the Successive Overrelaxation (SOR) method to enhance the convergence rate of the iterative process. The value of *ω* controls the extent of the correction applied at each step of the iteration:

• When 0<ω<1, the method performs *underrelaxation*, applying a smaller correction to the update. This can stabilize the process for certain ill-conditioned problems but may result in slower convergence.
• When 1<ω<2, the method performs *overrelaxation*, applying a larger correction to accelerate convergence. However, improper choice of *ω* in this range may lead to divergence.

The optimal value of *ω* depends on the spectral properties of the problem and is generally problem-specific. For the SOR-based iterative method presented here, *ω* is selected empirically or based on prior knowledge of the problem structure to achieve faster convergence while maintaining stability. The update formula,

X(k+1)=X(k)+ωH(k),

incorporates *ω* to scale the correction H(k), allowing for controlled progress toward the solution at each iteration.

Here is the modified algorithm incorporating the SOR method.

Method 3: Pure Newton method with Successive Overrelaxation - OSOR

1. Input a matrix $A\in R^{n\times n}$, an initial guess $X(0)\in R^{n\times n}$, and an overrelaxation parameter ω∈(0,2)∖{1}.
2. Solve for H(k) in the Lyapunov Eq. (5).
3. Update the iteration using:
  X(k+1)=X(k)+ωH(k)
  for all k=0,1,2,⋯.
4. Check if ${\left\|X(k+1)-X(k)\right\|}_{\text{F}}\leq \epsilon$. If true, stop; otherwise, go back to step 2.
5. Display the solution *X*, time in seconds and the residual ${\left\|F(X(k))\right\|}_{\text{F}}$.

## 4 Normwise, mixed and componentwise condition numbers

The sensitivity of solutions to perturbations in data is a critical concern in numerical analysis. In the context of the Yang-Baxter Equation (YBE), understanding how small changes in the input matrix affect the solution is essential for assessing the stability of computational algorithms. This section focuses on mixed and componentwise condition numbers, denoted as m(Φ) and c(Φ), which provide finer measures of sensitivity compared to the normwise condition number κ(Φ). By exploring these condition numbers, we gain insights into the localized and component-specific behavior of perturbations, offering a more nuanced understanding of solution stability.

Lemma 2 *Suppose that*M*is invertible and well-conditioned. The normwise, mixed, and componentwise condition numbers, denoted by*κ(Φ)*,*m(Φ)*, and*c(Φ)*respectively, satisfy the following relationships:*

(i) $\kappa (\Phi )=\frac{{\left\|M^{-1}\Lambda \right\|}_{2}{\left\|\Theta \right\|}_{2}}{{\left\|X\right\|}_{2}}$*,*
(ii) $m(\Phi )=\frac{{\left\|\left|M^{-1}\Lambda \right|\left|\Theta \right|\right\|}_{\infty }}{{\left\|X\right\|}_{\text{max}}}$*,*
(iii) $c(\Phi )={\left\|\frac{\left|M^{-1}\Lambda \right|\left|\Theta \right|}{\text{vec}\left(|X|\right)}\right\|}_{\infty }$*,*

where

$$M=\left((A^{T}⊗A)-(I⊗(XA))-({\left(AX\right)}^{T}⊗I)\right),\Theta =\text{vec}(A),\Lambda =\left((X^{T}⊗X)-(I⊗(AX))-({\left(XA\right)}^{T}⊗I)\right).$$

Proof Our focus is on deriving clear formulations for the normwise, mixed and componentwise condition numbers. To achieve this, we examine the perturbed equation:

(25) $$\overset{˜}{A}\overset{˜}{X}\overset{˜}{A}=\overset{˜}{X}\overset{˜}{A}\overset{˜}{X}.$$

Let us introduce small perturbations in the matrices *A* and *X* in Eq. (25):

(26) (A+δA)(X+δX)(A+δA)=(X+δX)(A+δA)(X+δX).

Expanding Eq. (26) yield Eq. (27):

(27) AXA+AXδA+AδXA+AδXδA+δAXA+δAXδA+δAδXA+δAδXδA=XAX+XAδX+XδAX+XδAδX+δXAX+δXAδX+δXδAX+δXδAδX.

Simplifying Eq. (27) leads to:

(28) $$A\delta XA-XA\delta X-\delta XAX=X\delta AX-AX\delta A-\delta AXA+O(\epsilon^{2}).$$

Now, considering vectorization in Eq. (28), we get:

(29) $$\begin{array}{c} (A^{T}⊗A)\mathrm{vec}(\delta X)-(I⊗(XA))\mathrm{vec}(\delta X)-({\left(AX\right)}^{T}⊗I)\mathrm{vec}(\delta X)=\\ (X^{T}⊗X)\mathrm{vec}(\delta A)-(I⊗(AX))\mathrm{vec}(\delta A)-({\left(XA\right)}^{T}⊗I)\mathrm{vec}(\delta A). \end{array}$$

Further simplification of Eq. (29) yields the following linear system:

(30) $$\begin{array}{c} \left((A^{T}⊗A)-(I⊗XA)-({\left(AX\right)}^{T}⊗I)\right)\mathrm{vec}(\delta X)=\\ \left((X^{T}⊗X)-(I⊗AX)-({\left(XA\right)}^{T}⊗I)\right)\mathrm{vec}(\delta A). \end{array}$$

Solving for vec(δX) in Eq. (30), we obtain:

$\mathrm{vec}(\delta X)=M^{-1}\Lambda \Theta$, where $M=\left((A^{T}⊗A)-(I⊗XA)-({\left(AX\right)}^{T}⊗I)\right)$, Θ=vec(δA), and $\Lambda =((X^{T}⊗X)-(I⊗AX)-({\left(XA\right)}^{T}⊗I))$.

Now, consider the map $\Phi :\Theta ={\left[\mathrm{vec}{\left(A\right)}^{T}\right]}^{T}\mapsto \mathrm{vec}(X)$. In line with the implicit function theorem, δX→0 as δA→0 because *δX* is a function of *δA*. We employ Lemma 1 to prove (i) and (ii) by examining the expressions for m(Φ) and c(Φ) and their components involving matrices M, Λ, Θ, and *X*. The specific structure of these matrices results in the established relations. The detailed derivation is omitted here for brevity. □

Furthermore, in our analysis, we establish two upper bounds for c(Φ) and m(Φ), each designed to provide understanding the behavior of Eq. (1). These upper bounds are defined as follows:

(i) $$\kappa (\Phi )\leq \frac{{\left\|M^{-1}\right\|}_{2}{\left\|\Lambda \right\|}_{2}{\left\|\Theta \right\|}_{2}}{{\left\|X\right\|}_{2}}=\kappa {\left(\Phi \right)}_{\text{Upper}},$$
(ii) $$m(\Phi )\leq \frac{{\left\|M^{{+}^{-1}}\right\|}_{\infty }{\left\|\Lambda^{+}\right\|}_{\infty }{\left\|\Theta^{+}\right\|}_{\infty }}{{\left\|X\right\|}_{\text{max}}}=m{\left(\Phi \right)}_{\text{Upper}},$$
(iii) $$c(\Phi )\leq {\left\|\frac{\left|M^{{+}^{-1}}||\Lambda^{+}||\Theta^{+}\right|}{\text{vec}(|X|)}\right\|}_{\infty }=c{\left(\Phi \right)}_{\text{Upper}},$$

where

$$M=\left((A^{T}⊗A)-(I⊗(XA))-({\left(AX\right)}^{T}⊗I)\right),\Theta =\text{vec}(A),\Lambda =(X^{T}⊗X)-(I⊗(AX))-({\left(XA\right)}^{T}⊗I),M^{+}=(|A{|}^{T}⊗|A|)+(|I|⊗(|X||A|))+({\left(|A||X|\right)}^{T}⊗|I|),\Theta^{+}=\text{vec}(|A|),\Lambda^{+}=(|X{|}^{T}⊗|X|)+(|I|⊗(|A||X|))+({\left(|X||A|\right)}^{T}⊗|I|).$$

Consider a perturbation *δA* on a matrix *A* such that |δA|≤ϵ|A|, where *ϵ* is a small positive parameter. We aim to derive local perturbation bounds: normwise, mixed, and componentwise for a matrix *X*.

1. Normwise perturbation bound:
  $$\frac{{\left\|\delta X\right\|}_{F}}{{\left\|X\right\|}_{F}}\leq \epsilon \kappa (\Phi ),$$
  where κ(Φ) is the condition number associated with the perturbation matrix Φ.
2. Mixed perturbation bound:
  $$\frac{{\left\|\delta X\right\|}_{\text{max}}}{{\left\|X\right\|}_{\text{max}}}\leq \epsilon m(\Phi ),$$
  where ${\left\|\delta X\right\|}_{\text{max}}$ and ${\left\|X\right\|}_{\text{max}}$ denote the maximum norm of *δX* and *X*, respectively, and m(Φ) captures the mixed characteristics of Φ.
3. Componentwise perturbation bound:
  $${\left\|\frac{\delta X}{X}\right\|}_{\text{max}}\leq \epsilon c(\Phi ),$$
  where ${\left\|\frac{\delta X}{X}\right\|}_{\text{max}}$ represents the maximum norm of the element-wise ratio of *δX* to *X*, and c(Φ) is the componentwise condition measure.

These limits offer valuable understanding regarding how susceptible the matrix *X* is to perturbations and are crucial for assessing the stability and flexibility of numerical algorithms that utilize matrix operations.

## 5 Numerical tests

The purpose of this section is to validate the theoretical results and demonstrate the practical effectiveness of the proposed methods for solving the Yang-Baxter-like matrix equation (YBME). We conduct a series of numerical experiments using MATLAB to compare the performance of different iterative techniques, including the accelerated Newton Method with exact line searches and successive over-relaxation. We focus on three key aspects: assessing the rate at which each method converges to a solution (convergence speed), evaluating the robustness of solutions in presence of perturbations (numerical stability), and comparing the precision of solutions obtained by each method (accuracy). The numerical tests are designed to cover a range of scenarios, including well-conditioned and ill-conditioned problems, to provide a comprehensive evaluation of the methods. We also explore the impact of different initial guesses and parameter settings on the performance of proposed methods.

We now present the experimental setup, including the test matrices and parameter configurations. We then discuss the results and analyze the performance of each method in detail.

Example 5.1 [7] Consider the matrix *A*, a 5×5 diagonalizable matrix, and let its Jordan form be represented as $A=SJS^{-1}$, in which J=diag(m,m,m,n,n);m=3,n=2 and

$$S=\left(\begin{array}{ccccc} -7 & -1 & 4 & 9 & -1\\ 17 & 2 & -10 & -19 & 2\\ -8 & -1 & 5 & 9 & -1\\ -7 & 0 & 4 & 8 & -1\\ 5 & 0 & -3 & -6 & 1 \end{array}\right).$$

We employ Method 2 with initial guess $X(0)=I_{5}$ and the same *δA* as in Example 5.2 to solve, Eq. (1) and a summary of the findings is recorded in Table 1.

**Table 1:** Comparison of condition numbers for Example 5.1.

	*j* = 8	*j* = 10	*j* = 12
*m*(Φ)	0.999999951546919	1.000000081127	0.9999999992763
*m*(Φ)_Upper_	374585.066039998	374585.162721692	374585.163155282
*c*(Φ)	1.00000044507343	1.0000000840082	1.000000018225
*c*(Φ)_Upper_	64976823.7351898	64976830.1654984	64976830.1084861
*κ*(Φ)	333.269629559679	333.269630548476	333.269630527669
*κ*(Φ)_Upper_	139039.760602149	139039.786854556	139039.786981552
$\frac{{\left\|\delta X\right\|}_{F}}{{\left\|X\right\|}_{F}}$	2.94686632052705e-08	5.0622381963389e-10	2.7500271863417e-12
*ϵκ*(Φ)	2.94686632088027e-08	5.06223819634311e-10	2.7500271863417e-12
$\frac{{\left\|\delta X\right\|}_{\text{max}}}{{\left\|X\right\|}_{\text{max}}}$	3.41538679584142e-08	6.4150868543551e-10	3.4604540043642e-12
*ϵm*(Φ)	3.4153867962508e-08	6.41508685436044e-10	3.4604540043644e-12
${\left\|\frac{\delta X}{X}\right\|}_{\text{max}}$	1.04531364202537e-06	1.54689840325149e-08	1.16250236836086e-10
*ϵc*(Φ)	1.04531364215067e-06	1.54689840325277e-08	1.16250236836086e-10

In Table 1, as *j* increases, the values for error bounds tend to decrease, indicating improved precision in the computations. Additionally, it is noted that the relationship m(Φ)≤c(Φ), $c(\Phi )\leq c_{\text{Upper}}(\Phi )$, and $m_{\text{Upper}}(\Phi )\leq c_{\text{Upper}}(\Phi )$ persists consistently across variations in *j*.

Example 5.2 We investigate a matrix utilized in a model characterizing the population dynamics of the bilby, with a specific emphasis on the quasi-stationary behavior within quasi-birth-death processes. The bilby is an endangered Australian marsupial which is the focus of this population model [16]. Let $A_{2}$ be defined as Q(u,v), a 5×5 matrix:

$$A_{2}=Q(u,v)=\left(\begin{array}{ccccc} uv_{1} & (1-u)v_{1} & 0 & 0 & 0\\ uv_{2} & 0 & (1-u)v_{2} & 0 & 0\\ uv_{3} & 0 & 0 & (1-u)v_{3} & 0\\ uv_{4} & 0 & 0 & 0 & (1-u)v_{4}\\ uv_{5} & 0 & 0 & 0 & (1-u)v_{5} \end{array}\right).$$

**Figure 1:**
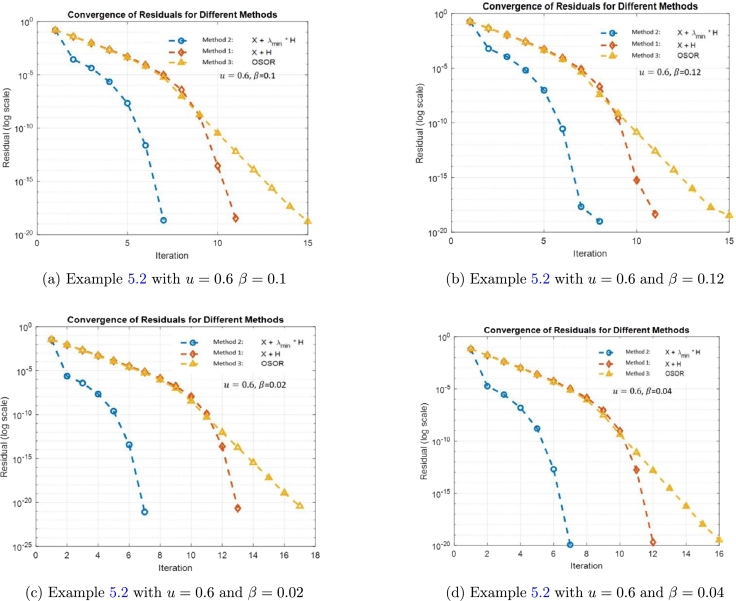
Convergence analysis of Methods 1-3 with different *β* values.

Here, the vector v=[0,0.5,0.55,0.8,1] represents the probabilities of the population moving down a level given phase *j*, and u=0.2. Now, considering β=0.5, matrix *B* is defined as $\beta A_{2}^{T}$. The symmetric matrix *A* is then formed using the equation $A=1/2(B+B^{T})$.

$$A=\frac{\beta }{2}\left(\begin{array}{ccccc} 2uv_{1} & uv_{2} & uv_{3} & uv_{4} & uv_{5}\\ uv_{2} & 2(1-u)v_{2} & 0 & 0 & 0\\ uv_{3} & 0 & 2(1-u)v_{3} & 0 & 0\\ uv_{4} & 0 & 0 & 2(1-u)v_{4} & 0\\ uv_{5} & 0 & 0 & 0 & 2(1-u)v_{5} \end{array}\right),$$

and $\delta A=rand(size(A))A10^{-j}$ and $\epsilon =\frac{\left\|\delta A\right\|}{\left\|A\right\|}$. We employ Method 2 with initial guess $X(0)=I_{5}$ to solve Eq. (1) and a summary of the findings is recorded in Fig. 1, Table 2 and Table 4, Table 5, Table 6, Table 7.

**Table 2:** Comparison of condition numbers for Example 5.2.

	*j* = 8	*j* = 10	*j* = 12
*m*(Φ)	0.999998896602141	0.99999993013415	0.99999999961547
*m*(Φ)_Upper_	163.290084309361	163.290192590244	163.290192801525
*c*(Φ)	0.999999067073763	0.99999993860773	0.99999999961547
*c*(Φ)_Upper_	6678.25009046028	6678.26302011466	6678.26303643559
*κ*(Φ)	1.32965326099088	1.32965378690765	1.32965378067915
*κ*(Φ)_Upper_	123.314047042244	123.314217174433	123.314217452282
$\frac{{\left\|\delta X\right\|}_{F}}{{\left\|X\right\|}_{F}}$	5.684311831334e-07	5.55822532458031e-09	5.5372509542184e-11
*ϵκ*(Φ)	5.6843118307321e-07	5.5582253569107e-09	5.5372509522745e-11
$\frac{{\left\|\delta X\right\|}_{\max}}{{\left\|X\right\|}_{\max}}$	6.2666583786354e-07	8.55631359577333e-09	6.8464957419002e-11
*ϵm*(Φ)	6.2666225503089e-07	8.5563136450509e-09	6.84649574146789e-11
${\left\|\frac{\delta X}{X}\right\|}_{\text{max}}$	1.1023000282489e-05	1.51417482192882e-07	1.38394879851499e-09
*ϵc*(Φ)	1.10230065613152e-05	1.51417483064921e-07	1.38394879859138e-09

**Figure 2:**
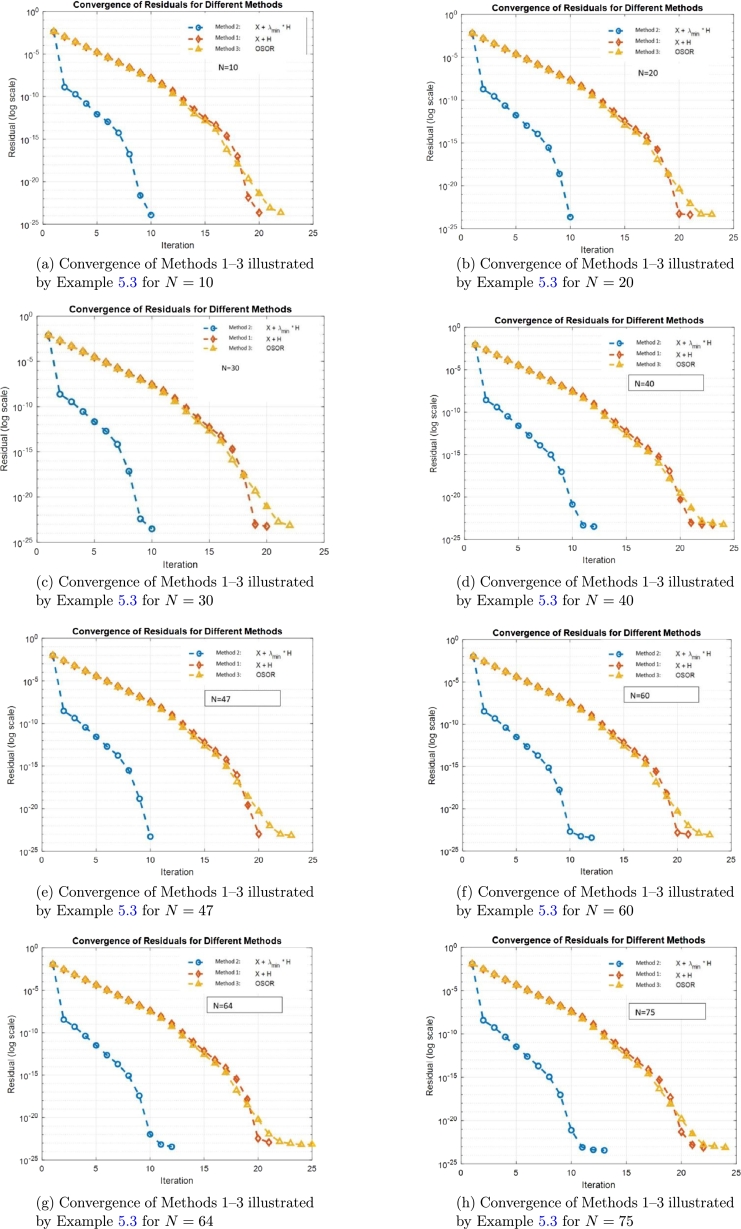
Convergence of Methods 1–3 using Example 5.3 across varying input matrix.

Example 5.3 We consider matrix

$$A=\frac{1}{100}\left(\text{diag}(\text{main}\_\text{diag})+\text{diag}(\text{super}\_\text{diag},1)+\text{diag}(\text{sub}\_\text{diag},-1)\right)\;\;and\;\;X(0)=I_{N},$$

where:

• *N*: Size of the matrix;
• a=0.02: Value on the main diagonal;
• b=0.1: Value on the superdiagonal;
• c=0.1: Value on the subdiagonal;
• $\text{main}\_\text{diag}=a\cdot 1_{N}$;
• $\text{super}\_\text{diag}=b\cdot 1_{N-1}$;
• $\text{sub}\_\text{diag}=c\cdot 1_{N-1}$.
  Methods 1–3 are employed to find the solvent of Eq. (1) with OSOR(ω=1.019). Also, the same *δA* as in Example 5.2 is used to compute the condition numbers. Then, the results summary is recorded in Table 3 for the condition numbers and the convergence is shown in Fig. 2 and Tables Table 8, Table 9, Table 10, Table 11, Table 12, Table 13, Table 14, Table 15.

**Table 3:** Comparison of condition numbers for Example 5.3.

	*j* = 8, *N* = 3	*j* = 10, *N* = 10	*j* = 12, *N* = 20
*m*(Φ)	0.999999997554155	0.99999997158546	0.999999997493561
*m*(Φ)_Upper_	63.9039719730187	632.924073139553	16643.585651813
*c*(Φ)	0.999999998115311	1.00000144538392	1.00006892255694
*c*(Φ)_Upper_	410.203579584038	902006.731213009	10344942111.5312
*κ*(Φ)	1.25744101369614	2.02432779247003	4.06624849612431
*κ*(Φ)_Upper_	81.9123921574163	1304.04706500653	70474.3016377724
$\frac{{\left\|\delta X\right\|}_{F}}{{\left\|X\right\|}_{F}}$	1.32203345572488e-09	2.2053259844914e-09	4.41133030402297e-09
*ϵκ*(Φ)	1.3220334373379e-09	2.2053259944467e-09	4.4113303873516e-09
$\frac{{\left\|\delta X\right\|}_{\max}}{{\left\|X\right\|}_{\max}}$	1.25984184180712e-09	3.4544622553385e-09	3.3418155135694e-08
*ϵm*(Φ)	1.2598418434598e-09	3.454462356851e-09	1.3418151469565e-08
${\left\|\frac{\delta X}{X}\right\|}_{\text{max}}$	6.14667406155686e-09	9.461035182642e-07	2.68457742921903e-04
*ϵc*(Φ)	6.1466740690648e-09	9.4610351814868e-07	2.68457745922863e-04

Example 5.4 [48], [49], [50], [51] Consider the spin-1 Heisenberg chain describes a quantum system where each particle at a site has spin quantum number S=1, with $m_{S}\in \{-1,0,1\}$ as the eigenvalues of $S_{z}$. The total Hilbert space for two neighboring spins is the tensor product $C^{3}⊗C^{3}=C^{9}$, spanned by the basis states $|m_{S}^{\left(1\right)},m_{S}^{\left(2\right)}\rangle$, where $m_{S}^{\left(1\right)},m_{S}^{\left(2\right)}\in \{-1,0,1\}$. The interaction between two spins can be described by an *R*-matrix, which satisfies the Yang-Baxter equation $R_{12}R_{13}R_{23}=R_{23}R_{13}R_{12}$, ensuring the consistency of multi-particle scattering processes and the integrability of the system. For a spin-1 Heisenberg model, the *R*-matrix is given by R=I+γP, where *I* is the 9×9 identity matrix, *P* is the permutation operator swapping two spins, and *γ* is a coupling parameter. In the basis |1,1〉,|1,0〉,|1,−1〉,|0,1〉,|0,0〉,|0,−1〉,|−1,1〉,|−1,0〉,|−1,−1〉, the *R*-matrix has the structure:

$$A=R=\left(\begin{array}{ccccccccc} 1+\gamma & 0 & 0 & 0 & 0 & 0 & 0 & 0 & 0\\ 0 & 1 & \gamma & 0 & 0 & 0 & 0 & 0 & 0\\ 0 & \gamma & 1 & 0 & 0 & 0 & 0 & 0 & 0\\ 0 & 0 & 0 & 1 & \gamma & 0 & 0 & 0 & 0\\ 0 & 0 & 0 & \gamma & 1+\gamma & 0 & 0 & 0 & 0\\ 0 & 0 & 0 & 0 & 0 & 1 & \gamma & 0 & 0\\ 0 & 0 & 0 & 0 & 0 & \gamma & 1 & 0 & 0\\ 0 & 0 & 0 & 0 & 0 & 0 & 0 & 1 & \gamma \\ 0 & 0 & 0 & 0 & 0 & 0 & 0 & \gamma & 1+\gamma \end{array}\right).$$

**Figure 3:**
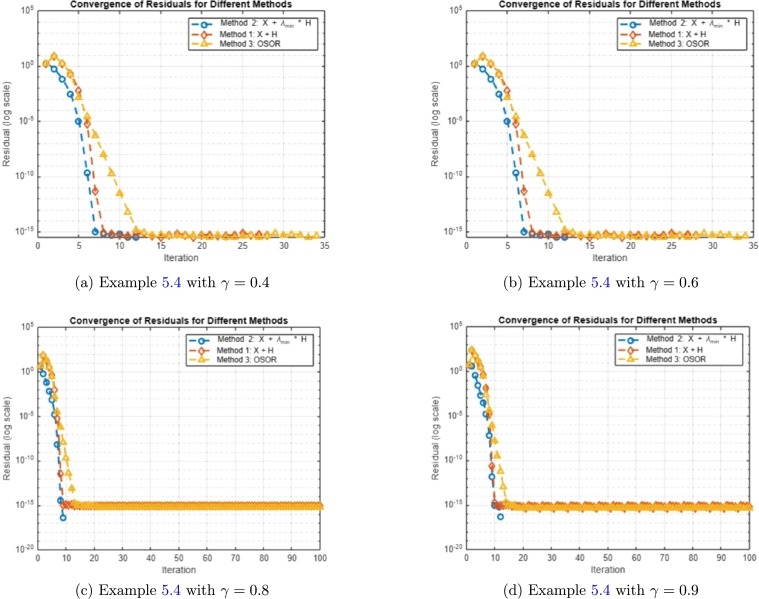
Convergence analysis of Methods 1-3 with different *γ* values.

Here, the diagonal terms 1+γ represent direct interactions between spins, while the off-diagonal terms represent spin-flip processes mediated by *γP*. The Yang-Baxter equation ensures that the interactions commute in a three-particle scattering scenario, enabling the system to remain integrable. This *R*-matrix encodes the spin exchange interactions from the spin-1 Heisenberg Hamiltonian $H=J\sum_{\langle i,j\rangle }S_{i}\cdot S_{j}$, where *J* is the coupling constant, and $S_{i}\cdot S_{j}$ includes spin exchange terms $S_{i}^{x}S_{j}^{x}+S_{i}^{y}S_{j}^{y}+S_{i}^{z}S_{j}^{z}$. We employ Methods 1–3 with initial guess $X_{0}=I_{9}$ to find the approximate solution of equation (1). A summary of results is presented in Fig. 3.

The results in Table 3 highlight the impact of increasing the matrix size *N* on the condition numbers and error metrics for Example 5.3. While the condition measures m(Φ) and c(Φ) remain close to 1, their corresponding upper bounds ($m{\left(\Phi \right)}_{\text{Upper }}$ and $c{\left(\Phi \right)}_{\text{Upper }}$) grow significantly with *N*. This indicates that the theoretical bounds on sensitivity increase as the problem size grows. Similarly, the condition number κ(Φ) shows a moderate increase with *N*, while its upper bound $\kappa {\left(\Phi \right)}_{\text{Upper }}$ grows substantially, reflecting greater potential sensitivity for large matrices. Despite these increases, the actual error metrics, including ${\left\|\delta X\right\|}_{F}/{\left\|X\right\|}_{F}$, ϵκ(Φ), and ${\left\|\delta X\right\|}_{\max}/{\left\|X\right\|}_{\max}$, remain small, indicating high accuracy and numerical stability.

In conclusion, while the complexity and potential error amplification increase with the size of the matrix *N*, the numerical results demonstrate that the proposed method maintains good accuracy and stability across varying problem sizes.

Based on Fig. 1(a) to 1(d), the convergence of Methods 1–3 improves as the value of *β* decreases from 0.1 to 0.02. Examining Table 4, Table 5, Table 6, Table 7, Method 2 exhibits the smallest residual, while Method 3 has the largest residual. In terms of computation time for the solution, Method 1 is the fastest, followed by Method 2. Overall, based on residual accuracy, Method 2 is the most accurate.

**Table 4:** Results for Example 5.2 with *u* = 0.6 and *β* = 0.1.

Method	Time_taken	Num_iterations	Final_residual
Method 2: *X* + *λ*_min_ ⋅ *H*	0.0110331	7	2.18947776865789 × 10^−19^
Method 1: *X* + *H*	0.0010166	11	3.3085935292859 × 10^−19^
Method 3: OSOR(*ω* = 1.019)	0.0011261	15	1.76182853028894 × 10^−19^

**Table 5:** Results summary for Example 5.2 with *u* = 0.6 and *β* = 0.12.

Method	Time_taken	Num_iterations	Final_residual
Method 2: *X* + *λ*_min_ ⋅ *H*	0.0112461	8	1.00165003645288 × 10^−19^
Method 1: *X* + *H*	0.001033	11	4.47848996436358 × 10^−19^
Method 3: OSOR(*ω* = 1.019)	0.0011553	15	3.43924583377654 × 10^−19^

**Table 6:** Results summary for Example 5.2 with *u* = 0.6 and *β* = 0.02.

Method	Time_taken	Num_iterations	Final_residual
Method 2: *X* + *λ*_min_ ⋅ *H*	0.0093413	7	8.32010481530407 × 10^−22^
Method 1: *X* + *H*	0.001063	13	2.22598099045175 × 10^−21^
Method 3: OSOR(*ω* = 1.019)	0.0011417	17	3.80723407672498 × 10^−21^

**Table 7:** Results summary for Example 5.2 with *u* = 0.6 and *β* = 0.04.

Method	Time_taken	Num_iterations	Final_residual
Method 2: *X* + *λ*_min_ ⋅ *H*	0.0094223	7	1.15519892379876 × 10^−20^
Method 1: *X* + *H*	0.0009406	12	2.01337433121584 × 10^−20^
Method 3: OSOR(*ω* = 1.019)	0.0010182	16	3.26519940158205 × 10^−20^

The data presented in Table 4, Table 5, Table 6, Table 7 indicate that Method 2 consistently achieves the smallest final residual, highlighting its robustness across all cases as *β* varies. In contrast, Method 1 emerges as the fastest in most scenarios, albeit with slightly larger residuals. Method 3, while requiring more iterations and computational time, still manages to produce relatively small residuals. These findings underscore the trade-off between speed and accuracy, with Method 2 excelling in residual minimization across all tested conditions.

The results across Table 8, Table 9, Table 10, Table 11, Table 12, Table 13, Table 14, Table 15 and Fig. 2(a) to 2(h) demonstrate that Method 2 consistently outperforms the other methods in terms of computational efficiency and convergence speed. For all tested values of *N*, Method 2 requires the least time and the fewest iterations (10 -13) while maintaining high accuracy, with final residuals typically on the order of $10^{-24}$. In contrast, Method 1 and Method 3 require more iterations (20 - 25) and take longer to converge, particularly as *N* increases. Despite slight differences in residuals, all methods achieve comparable accuracy. Overall, Method 2 is the most effective, especially for larger problem sizes.

**Table 8:** Results summary for Example 5.3 when *N* = 10.

Method	Time_taken	Num_iterations	Final_residual
Method 2: *X* + *λ*_min_⁎*H*	0.0439595	10	1.2123845847428e-24
Method 1: *X* + *H*	0.0152247	20	2.29406077155547e-24
Method 3: OSOR(*ω* = 1.019)	0.0158327	22	2.35480169276958e-24

**Table 9:** Results summary for Example 5.3 when *N* = 20.

Method	Time_taken	Num_iterations	Final_residual
Method 2: *X* + *λ*_min_⁎*H*	0.0643721	10	2.25270477126132e-24
Method 1: *X* + *H*	0.1985034	21	4.24917150523188e-24
Method 3: OSOR(*ω* = 1.019)	0.1879016	23	4.59115495143245e-24

**Table 10:** Results summary for Example 5.3 when *N* = 30.

Method	Time_taken	Num_iterations	Final_residual
Method 2: *X* + *λ*_min_⁎*H*	0.1985601	10	3.07170586962295e-24
Method 1: *X* + *H*	0.4114396	20	5.86207476159605e-24
Method 3: OSOR(*ω* = 1.019)	0.5341056	22	7.25352684132059e-24

**Table 11:** Results summary for Example 5.3 when *N* = 40.

Method	Time_taken	Num_iterations	Final_residual
Method 2: *X* + *λ*_min_⁎*H*	0.9280591	12	3.34442278361499e-24
Method 1: *X* + *H*	1.7869855	23	5.50732410583424e-24
Method 3: OSOR(*ω* = 1.019)	1.6959851	24	5.76594472254993e-24

**Table 12:** Results summary for Example 5.3 when *N* = 47.

Method	Time_taken	Num_iterations	Final_residual
Method 2: *X* + *λ*_min_⁎*H*	1.4547667	10	5.3672140718696e-24
Method 1: *X* + *H*	2.8151499	20	1.08382780468081e-23
Method 3: OSOR(*ω* = 1.019)	3.320381	23	7.34532982089198e-24

**Table 13:** Results summary for Example 5.3 when *N* = 60.

Method	Time_taken	Num_iterations	Final_residual
Method 2: *X* + *λ*_min_⁎*H*	9.8362336	12	3.83536699864688e-24
Method 1: *X* + *H*	14.6670614	21	9.68105649755148e-24
Method 3: OSOR(*ω* = 1.019)	15.9924161	23	7.87589957645125e-24

**Table 14:** Results summary for Example 5.3 when *N* = 64.

Method	Time_taken	Num_iterations	Final_residual
Method 2: *X* + *λ*_min_⁎*H*	6.1613177	12	3.68894656707277e-24
Method 1: *X* + *H*	11.0739327	21	1.21374023462537e-23
Method 3: OSOR(*ω* = 1.019)	13.4747626	25	7.12933592729118e-24

**Table 15:** Results summary for Example 5.3 when *N* = 75.

Method	Time_taken	Num_iterations	Final_residual
Method 2: *X* + *λ*_min_⁎*H*	13.1249311	13	3.73921450468443e-24
Method 1: *X* + *H*	24.3009462	22	7.86886454505175e-24
Method 3: OSOR(*ω* = 1.019)	27.45982	24	7.73687597619106e-24

Based on Fig. 3(a) to 3(d), the convergence analysis reveals that Method 2 exhibits superior performance across all tested values of *γ* (γ=0.4,0.6,0.8,0.9). Method 2 demonstrates excellent stability, as it converges consistently without oscillations or irregular behavior in the residuals. In terms of accuracy, Method 2 achieves small residuals across all *γ* values, consistently meeting the prescribed tolerance of $1.0\times 10^{-16}$. Furthermore, Method 2 converges faster than the other methods, requiring significantly fewer iterations than the maximum allowed (100) and consistently outpacing Methods 1 and 3. For smaller values of *γ* (γ=0.4 and γ=0.6), Method 2 reaches the solution in fewer iterations while achieving the smallest residuals. For larger values of *γ* (γ=0.8 and γ=0.9), Method 2 continues to outperform Method 1, which converges more slowly, and Method 3, which, although accurate, requires more iterations. The efficiency and accuracy of Method 2 make it the most robust and reliable method for all tested cases, particularly when rapid convergence and minimal residuals are desired. Method 1 performs adequately for smaller *γ* values but struggles to maintain efficiency as *γ* increases. Method 3 is stable and accurate for higher *γ* values but generally requires more iterations than Method 2. Overall, Method 2 is recommended for its quick convergence, small residuals, and efficiency across a wide range of coupling strengths.

## 6 Conclusion

This paper presents the exact line search and successive overrelaxation methods to enhance the convergence of the Newton method for solving the Yang-Baxter matrix equation. Additionally, the normwise, mixed, and componentwise condition numbers, along with their upper bounds, are derived. Numerical experiments demonstrate that the exact line search technique improves the convergence of the Newton method, particularly in terms of computational time for larger matrix sizes, while reducing the number of iterations across all numerical examples considered. It also produces a smaller residual compared to the successive overrelaxation method. Regarding the condition numbers, the mixed condition numbers for all problems considered were close to one, suggesting that the problems are well-conditioned. However, the normwise condition numbers were relatively large, indicating potential sensitivity to perturbations in the matrix.

## CRediT authorship contribution statement

**Chacha Stephen Chacha:** Writing – review & editing, Writing – original draft, Visualization, Validation, Supervision, Software, Project administration, Methodology, Investigation, Formal analysis, Data curation, Conceptualization.

## Declaration of Competing Interest

The author declares that there is no conflict of interest regarding the publication of this paper. The research and findings presented in this study were conducted in an impartial and unbiased manner, with no financial, personal, or professional relationships that could have influenced the outcomes or interpretations of the results.

## Data Availability

All data supporting the findings of this study are provided within the manuscript. Code used for the research described in the article will be made available upon request. For code requests, please contact the corresponding author.
